# Determinants of puerperal sepsis among postpartum women at public hospital in Gedeo Zone, southern Ethiopia, 2023: unmatched case–control study

**DOI:** 10.1186/s12884-025-08626-5

**Published:** 2026-01-09

**Authors:** Mulugeta Edao Shate, Birhanu Teshome, Edao Sinba Etu, Bikila Lencha

**Affiliations:** Department of Epidemiology, School of Public Health, College of Health Sciences, Madda Walabu University, P.O. Box 238, Shashemene, Ethiopia

**Keywords:** Puerperal sepsis, Postpartum women, Determinant, Case–control studies, Ethiopia

## Abstract

**Introduction:**

The World Health Organization records 295,000 maternal deaths during childbirth each year, of which 15% are due to postpartum sepsis. In Ethiopia, it is the third cause of maternal death after hemorrhage and hypertension. However, data on the determinants of puerperal sepsis in the study area are scarce.

**Objective:**

This study aimed to identify determinants of puerperal sepsis among postpartum women at a public hospital in Gedeo Zone, southern Ethiopia, in 2023.

**Methods:**

A hospital-based, unmatched case–control study was conducted among 475 postpartum women (119 cases and 356 controls with a ratio of 1:3) at public hospitals in the Gedeo Zone from July 1 to September 20, 2023. All cases were included, and Systematic random sampling was employed to select controls. A pretested and structured face-to-face interview questionnaire was conducted. Data were entered, cleared, and edited using EPI-data software 4.6 and then exported to Statistical Package for Social Science version 26 for analysis. Binary logistic analysis was executed, and all variables with a p-value of < 0.25 were entered into multivariable logistic regression. Finally, multivariable logistic regression analysis was employed to identify independent determinants of puerperal sepsis. A *P*-value of > 0.05 at the 95% CI was considered the cut-off point to declare a statistically significant association.

**Result:**

Four hundred seventy-five (119 cases and 356 controls) were included in this study with a 98.96% response rate. Variables identified as significantly associated with puerperal sepsis were no ANC follow-up (AOR = 4.19, 95% CI (2.32, 7.58), cesarean section (AOR = 2.74, 95% CI (1.19, 6.31), postpartum hemorrhage (AOR = 2.57, 95% CI (1.39, 4.74), duration of rupture of membrane ≥ 24 h (AOR = 3.31, 95% CI(1.89, 5.83), duration labor ≥ 24 h (AOR = 3.15 95% CI(1.09, 9.43), and anemia (AOR = 2.46, 95% CI (1.39, 4.32).

**Conclusion:**

In this study, variables significantly associated with puerperal sepsis were the absence of antenatal care, cesarean section, postpartum hemorrhage, prolonged PROM, duration of labor ≥ 24 h, and anemia. Puerperal sepsis associated with these factors should be minimized by following labor management protocol and emphasizing information on dietary diversification.

## Introduction

Puerperal sepsis is defined as an infection of the genital tract that can develop up to six weeks after delivery and can happen at any point following the rupture of the membranes. It is accompanied by two or more of the following symptoms: pelvic pains, elevated body temperature (38.5 °C), atypical vaginal discharge, unpleasant discharge odor, and a delay in the uterus's size decrease (2 cm per day in the first eight days) [1].

The World Health Organization (WHO) reported that puerperal sepsis accounts for 15% of the approximately 295,000 maternal fatalities that occur each year during labor and delivery [2]. An estimated 62,000 maternal fatalities are attributed to maternal sepsis, with up to 5.2 million new cases reported each year [3]. The major causes of puerperal sepsis are bilateral tubal blockage, infertility, acute or chronic pelvic inflammatory illness, and maternal fatalities [4]. Puerperal sepsis is among the leading causes of preventable maternal morbidity and mortality, not only in developing countries but also in developed countries, which account for 10.7% and 4.7% of maternal deaths, respectively [5]. It is placed sixth among women of reproductive age in terms of illness burden, depression, HIV/AIDS, tuberculosis, abortion, and schizophrenia [3].

In Sub-Saharan Africa, maternal mortality due to puerperal sepsis increased from 9.7% in 2009 [6] to 10.3% in 2014 [5]. Puerperal sepsis was the cause of maternal mortality in retrospective research from Mbarara, Uganda, accounting for 30.9% of maternal deaths. Postpartum hemorrhage came in second at 21.6% [7]. A study conducted in Tanzania showed that the prevalence of puerperal sepsis was 11.5% [8].

In Ethiopia, puerperal sepsis is the third direct primary cause of maternal death after hemorrhage and hypertension [9, 10]. According to a Gonder Referral Hospital study, puerperal sepsis occurred in 17.2% of women during childbirth and the postpartum period [11]. Puerperal sepsis was the fourth most common cause of maternal deaths in Ethiopia, accounting for 13% of all maternal deaths, according to another study on maternal mortality [12].

The researchers identified several risk factors associated with puerperal sepsis, including age > 30 years, lack of antenatal care follow-up, mode of placental removal, a referral from other health institutions, anemia, delivery by cesarean section, premature rupture of the membrane, prolonged labor, frequent vaginal examinations with poor hygiene and poor aseptic technique, postpartum hemorrhage, lack of formal education, and low income [13, 14].

To prevent infections related to labor and delivery, the WHO technical group suggests using antibiotics for cesarean section (C/S), PPROM, third- or fourth-degree perineal tears, manual placenta removal, and operative vaginal births; additionally, antimicrobial use during vaginal and C/S deliveries and per-vaginal examination should be performed every four hours [15]. Similarly, the World Health Organization suggests that to lower the number of maternal mortalities, no woman in a health facility should develop puerperal sepsis [16].

Despite this, puerperal sepsis is one of the causes of maternal death in Ethiopia, which ranks among the nations with the highest rates of maternal mortality. At an estimated 412 deaths per 100,000 live births, the maternal mortality rate is still high, according to the Ethiopian Demographic and Health Survey 2016 (EDHS) [17]. As part of the Sustainable Development Goals (SDGs), every country should reduce the maternal mortality ratio to 70 per 100,000 by 2030 [18]. Ethiopia's Health Sector Transformation Plan (HSTP II) aims to reduce the country's maternal mortality rate from 401 per 100,000 live births to 279 per 100,000 live births between 2020–2021 and 2024–2025 [19].

To reach our country's SDG and HSTP II plan targets, it is better to identify the causes and contributing factors for developing puerperal sepsis, which is very important to reduce maternal morbidity and mortality. Although puerperal sepsis is among the leading causes of maternal mortality, there has been a scarcity of studies conducted to investigate the determinants of puerperal sepsis in this study area. Furthermore, this study incorporates additional variables not previously examined in Ethiopia. Previous research conducted in other countries has linked obstructed labor, multiple pregnancies, and post-term pregnancy to cases of puerperal sepsis [20–23]. Therefore, this study aimed to identify determinants of puerperal sepsis among postpartum women who received services at public hospitals in the Gedeo Zone.

## Methods and materials

### Study area and period

A study was conducted in Gedeo Zone in four Government hospitals from July 1 to September 20, 2023. Gedeo Zone is one of the zones found in the Southern Nations, Nationalities, and Peoples Region (SNNPR), with the administrative center of Dilla Town located 370 km from Addis Ababa and 86 km from the regional city Hawassa. Based on the 2022 annual report of the Gedeo Zone health department, the estimated number of mothers of reproductive age is 256,487, and the estimated number of deliveries is 43,178. In the Gedeo Zone, the skilled delivery rate is 50%. The zonal coverage for ANC 4 was 58%, and postnatal care was 80%. The zone has one referral teaching hospital, three primary hospitals, 38 health centers, 149 health posts, nine nongovernmental organizations, one private hospital, and more than 27 private clinics. All hospitals provide comprehensive emergency obstetric care services [24].

### Study design

The study design was a hospital-based, unmatched case–control study design.

### Population of the study

#### Source population

All postpartum women received services at a public hospital in the Gedeo Zone within 42 days of delivery.

#### Study population

*Cases*: All postpartum women who received services at a public hospital in Gedeo Zone within 42 days of delivery were diagnosed with puerperal sepsis during the data collection period.

*Controls*: postpartum women who received services at a public hospital in Gedeo Zone within 42 days of delivery without puerperal sepsis were selected during the data collection period.

### Eligibility Criteria

#### Inclusion criteria for cases

Postpartum women received services at a public hospital in Gedeo Zone within 42 days of delivery from July 1 to September 20, 2023, diagnosed with puerperal sepsis.

#### Inclusion criteria for controls

Postpartum women who received services at a public hospital in Gedeo Zone within 42 days of delivery from July 1 to September 20, 2023, were not diagnosed with puerperal sepsis.

#### Exclusion criteria for cases

Postpartum women, those who were in the intensive care unit to the extent that they were unable to communicate until the end of the study period, and women with diseases such as hepatitis infection and malaria, were excluded from the study. Those selected participants came the second time to avoid repetition.

#### Exclusion criteria for controls

Those who were unable to speak, hear, or come for the second time in the study period.

### Sample size and sampling methods

#### Sample size determination

The sample size was determined using an unmatched case–control study in EPI Info software version 7. Considering the assumption that the ratio of cases to controls was 1:3, 95% CI, 80% power of the study, the AOR was 3.43, and the proportion of exposed controls was 4.13%. Taking the prolonged labor associated with puerperal sepsis from the recent study conducted in Hawassa City [23], was given 436 total sample size (109 cases and 327 controls), and by adding a 10% non-response rate, the final sample size was 480 (120 cases and 360 controls) (Table 1).

**Table 1:** Sample size determination for determinants of puerperal sepsis among postpartum women at Public Hospital in Gedeo Zone, Southern Ethiopia, 2023

S. No	Variables	% control exposed	AOR	Ratio	Sample size with a 10% non-response rate	Reference
1	Duration of PROM	16	3.73	1:3	154	[25]
2	Rural residence	28.6	2.5	1:3	260	
3	Delivery by cesarean section	16.4	3.5	1:3	250	[23]
4	Prolonged labor	4.13	3.43	1:3	**480**	

#### Sampling technique

**Fig. 1:**
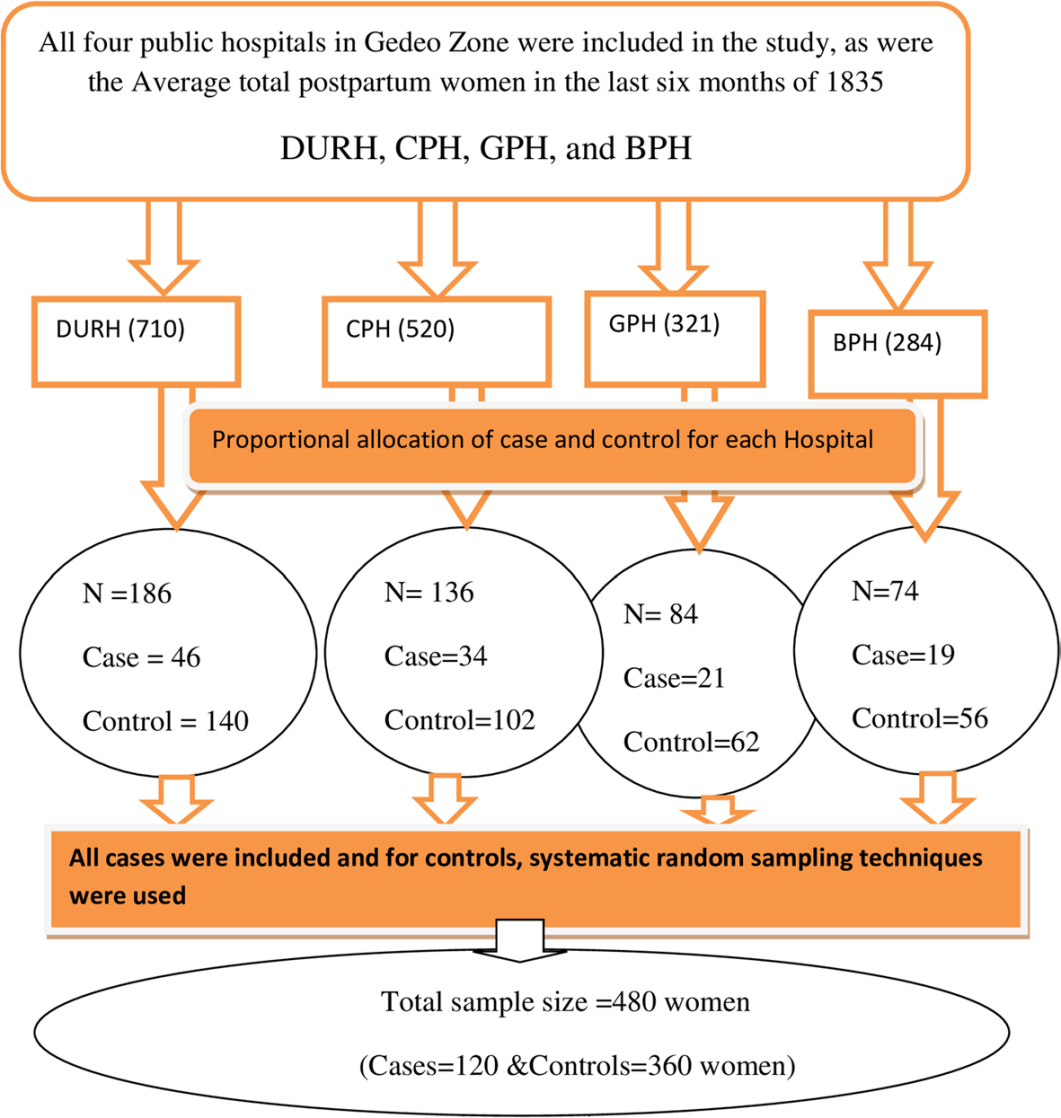
Schematic presentation of the sampling procedure for determinants of puerperal sepsis among postpartum women at a public hospital, in Gedeo Zone

All four public hospitals in the Gedeo Zone were included in the study. The sample size was proportionally allocated to each hospital based on the client flow in the previous three months. All women diagnosed with puerperal sepsis by physicians during the data collection period in each hospital were selected as cases (due to the rare cases). Women without puerperal sepsis who met the inclusion criteria were selected using systematic random sampling techniques as controls and interviewed during the study period. Based on the average number of postpartum women who were getting postnatal care service (admitted to the gynecology ward and getting postnatal care service) in four hospitals in the previous six months reported, an average of 1689 nonpuerperal sepsis postpartum women and 146 were diagnosed with puerperal sepsis. The expected numbers for each hospital were as follows: Dilla University Referral Hospital (DURH) was 64, Cheffe Primary Hospital (CPH) was 38, Gedeb Primary Hospital (GPH) was 26, and Bule Primary Hospital (BPH) was 18. All postpartum women diagnosed with puerperal sepsis were selected as cases until the required sample size was achieved, and three controls for each case were selected using systematic sampling. The first Control women were selected randomly, then continued every 5th interval since k = 1689/360 = 5 (Fig. 1).

### Study variables

#### Dependent variable

Puerperal sepsis is a dummy variable that takes a value of one if the respondent has the disease and zero otherwise.

### Independent variables

#### Socio-demographic characteristics

Age, Residence, educational status, maternal occupation.

#### Obstetrics factors

Parity, multiple pregnancies, Mode of delivery, prolonged labor, Premature Rupture of Membrane, frequent vaginal examination, Hx of ANC follow-up, preeclampsia, postpartum hemorrhage, obstructed labor, and Post-term birth.

#### Maternal Medical factors

Gestational diabetes mellitus, Anemia, sexually transmitted infection, and HIV status.

*Health-related factors*: referral status, Hygienic practice, and Attendant during delivery.

### Operational and term definitions

Puerperal sepsis is an infection of the genital tract that can happen at any point between the 24-h mark following birth and the 42nd day postpartum, and it is characterized by the presence of two or more of the following symptoms: pelvic pain, Fever, defined as a fever of 38 °C or above at any time, pungent vaginal discharge, and a delay in the uterus's pace of shrinkage (less than 2 cm/day for the first eight days) [1].

#### Cases

Postpartum mothers admitted to the ward or coming to the postnatal clinic at the selected hospitals during the study period who were diagnosed with puerperal sepsis by physicians working in maternity wards selected as cases [16].

#### Controls

Postpartum mothers admitted to or coming to the postnatal clinic in the selected hospitals during the study period who had no puerperal sepsis in their medical records were enrolled as controls. As soon as the cases were diagnosed, three controls were chosen on the same day from the same hospital for each case.

*Prolonged premature rupture of membrane*: the amniotic membrane ruptures for longer than 24 h.

*Prolonged labor*: in cases where labor lasts more than 24 h, as the start of regular, rhythmic, painful contractions followed by cervical dilatation [26].

*Gestational diabetes mellitus*: postpartum women were considered as having GDM when their fasting plasma glucose was ≥ 126 mg/dl, two-hour plasma glucose after taking 75 g oral glucose, and random plasma glucose was ≥ 200 mg/dl with the presence of symptoms of DM, and were taken from their medical cards [27].

*Anemia*: postpartum women were considered as having Anemia; their hemoglobin level of < 11 g/dl was taken from their medical cards [28, 29].

### Data collection methods

A structured, face-to-face interview questionnaire was adapted from different literature and was used to collect data from the study participants [30, 31]. The questionnaire was written in the English language following a thorough assessment of previously verified published research. Then, the questionnaire was translated into Amharic, and the data collector used the Amharic version of the questionnaire to collect data from the study participants. Again, it was translated back into English for analysis. Data were collected by ten midwives and supervised by two health Officers. Furthermore, patient cards were reviewed to collect information on obstetric and medical characteristics, and data on maternal socio-demographic characteristics and other factors that couldn't be obtained from their medical cards were collected from the women by using an interviewer.

### Data quality management

Data collectors and supervisors were trained for two days in data-gathering processes. The questionnaire was designed and written in English first, then translated into Amharic by language experts, and finally back to English to ensure consistency. The data was collected in the Amharic version. Before the data was collected, a pre-test was done on 5% (6 cases and 18 controls) of the postpartum mothers admitted to Chuko Hospital. The daily investigator evaluated and checked the obtained data for completeness and consistency. The internal consistency was verified by Cronbach's alpha, which was 0.82, showing good reliability.

#### Data management and analysis

Data were coded, cleaned, and entered into EpiData 4.6 and then exported to SPSS version 26 for analysis. Descriptive statistics such as frequencies and percentages were used to summarize the variables. Bivariate logistic regression was first done to assess the association of each independent variable with puerperal sepsis. In the multivariable model, variables with a p-value less than 0.25 in bivariable analysis were fitted. An adjusted odds ratio (AOR) with 95% CI was computed to ascertain the strength of association, and the level of statistical significance was considered at p < 0.05.

The Hosmer–Lemeshow goodness-of-fit test was conducted to confirm the fitness of the model, which was adequate, with a p-value of 0.306. VIF and tolerance values were also measured to check on multicollinearity; none of these were significant. Therefore, the results have been presented using adjusted odds ratios along with their respective 95% CI. 

#### Dissemination plan

Finally, the findings of the study were submitted to Madda Walabu University, Shashemene Campus, and the Public Health Department of Postgraduate Study. It will be presented at the last defense. It will also be communicated to the Gedeo Zone health department. Further efforts will be made to publish in national and international peer-reviewed journals.

## Result

### Socio-demographic characteristics

Out of the total sample size of 480, 475 postpartum women (119 cases and 356 controls) were included in the analysis, resulting in a response rate of 98.96%. The age of respondents ranges from 16 to 48 years, with a mean age of 28.21 ± 5.954 for both case and control groups. Thirty-seven percent of cases and 111 (31.92%) of the control respondents were in the age group of 25–31 years. The majority of the control group, 207 (58.1%), and 50 (42.0%) of the cases were urban residents. Concerning educational status, 48 (40.3%) of cases and 98 (27.5%) of controls had no formal education. Regarding occupational status, 57 (49.9%) of the cases and 134 (37.6%) of the control group were housewives (Table 2).

**Table 2:** Socio-demographic characteristics of respondents on postpartum mothers at a public hospital in Gedeo zone, 2023

Variable		Puerperal sepsis status		Total N (%)
		Case N (%)	Control N (%)	
Age	16–25	36 (30.3)	128 (36.0)	164 (34.5)
	25–31	44 (37.0)	111 (31.2)	155 (32.6)
	≥ 32	39 (32.8.9)	117(32.9)	156 (32.8)
Residence	Urban	50 (42.0)	207 (58.1)	257 (54.1)
	Rural	69 (58.0)	149 (41.9)	218 (45.9)
Mother’s education	No formal education	48 (40.3)	98 (27.5)	146 (30.7)
	Primary	26 (21.8)	104 (29.2)	130 (27.4)
	Secondary	23 (19.3)	92 (25.8)	115 (24.2)
	College and above	22 (18.5)	62 (17.4)	84 (17.7)
The educational status of her husband	No formal education	33 (31.1)	95 (26.7)	132 (28.4)
	Primary	35 (29.4)	106 (29.8)	141 (29.7)
	Secondary	31 (26.1)	95 (26.7)	126 (26.5)
	College and above	16 (13.4)	60 (16.9)	76 (16.0)
Marital Occupation	House-wife	57 (49.9)	134 (37.6)	191 (40.2)
	Self-employed	31 (26.1)	101 (28.4)	132 (27.8)
	Government employer	21 (17.6)	78 (21.9)	99 (20.8)
	others*	10 (8.4)	43 (12.1)	53 (11.2)

^*^merchant, daily laborer, farmer, and student

### Obstetric characteristics

With regards to ANC follow-up, 68 (57.1%) cases and 80 (22.5%) controls were reported as having no ANC follow-up. Among the participants, 28 (23.5%) cases and 74 (20.8%) controls were Primiparae. Almost all, 311 (87.4%), controls were delivered in health institutions. Forty-eight (40.3%) cases and 58 (16.3%) controls had a postpartum hemorrhage. Among the mothers, 18 (15.1%) cases had multiple pregnancies.

The majority of participants in the study (72.3% cases, 82.5% controls) had term gestation, and episiotomies were performed on only 31 (26.1%) of cases and 78 (21.9%) of controls.

Of the postpartum mothers included in the study, 70 (58.8%) of the cases and 75 (21.1%) of the controls had a Duration of labor ≥ 24 h (Table 3).

**Table 3:** Obstetric characteristics of postpartum women at Gedeo zone public hospitals, Southern Ethiopia, 2023

variable		Puerperal sepsis status		Total N (%)
		Case N (%)	Control N (%)	
Parity	Primipara	28 (23.5)	74 (20.8)	102 (21.5)
	Multipara	55 (46.2)	195 (54.8)	250 (52.6)
	Grand multipara	36 (30.3)	87 (24.4)	123 (25.9)
ANC follow-up	Yes	51 (42.9)	276 (77.5)	327 (68.8)
	No	68 (57.1)	80 (22.5)	148 (31.2)
Number of ANC follow-ups	≤ 2 times	24 (51.1)	148 (53.6)	172 (53.3)
	≥ 3 times	23 (48.9)	128 (46.4)	151 (46.7)
Place of delivery	Home	29(24.4)	45 (12.6)	74 (15.6)
	Health institutions	90 (75.6)	311 (87.4)	401 (87.4)
Prophylaxis antibiotic used	no	86 (72.3)	275 (77.2)	361 (76.0)
	yes	33 (27.7)	81 (22.8)	114 (24.0)
Gestational age	Preterm	12 (10.1)	22 (6.2)	34(7.2)
	Term	86 (72.3)	193(82.5)	378 (79.6)
	post term	21 (17.6)	42 (11.8)	63 (13.3)
multiple pregnancies	Yes	18(15.1)	46(12.9)	64 (13.5)
	No	101 (84.9)	310 (87.1)	411 (86.5)
obstructed labor	Yes	19 (16.0)	29 (8.1)	48 (10.1)
	No	100 (84.0)	327 (91.9)	427(89.9)
Duration of labor	< 12 h	6 (5.0)	31 (8.7)	37 (7.8)
	12–24 h	43 (36.1)	250 (70.2)	293 (61.7)
	≥ 24 h	70 (58.8)	75 (21.1)	145 (30.5)
labor started	Spontaneous	95 (79.8)	298 (83.7)	393(82.7)
	Induction	24 (20.2)	58 (16.3)	82 (17.3)
Episiotomy done	Yes	31(26.1)	78 (21.9)	109 (22.9)
	No	88 (73.9)	278 (78.1)	366 (77.1)
PPH	yes	48 (40.3)	58 (16.3)	106(22.3)
	No	71 (59.7)	298 (83.7)	369 (77.7)
preeclampsia	Yes	31 (26.1)	65 (18.3)	96 (20.2)
	No	88 (73.9)	291 (81.3)	379 (79.8)

Abbreviation: *ANC* Antenatal care, *PPH* Postpartum hemorrhage

**Fig. 2:**
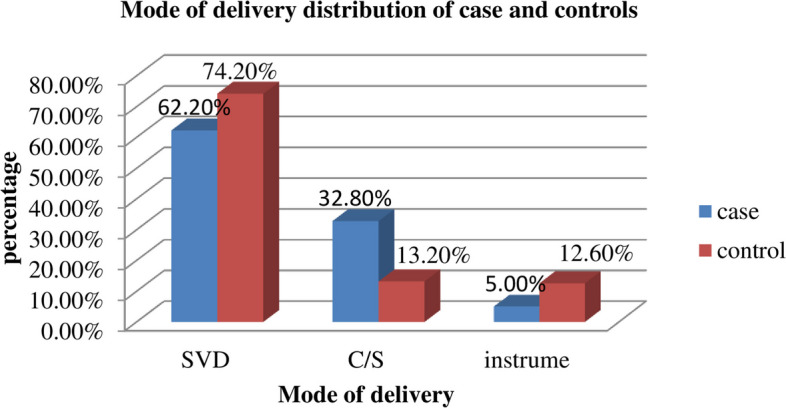
Mode of delivery of postpartum women at Gedeo Zone public hospitals, Southern Ethiopia, 2023

Among the postpartum women included in the study, 74 (62.2%) of the cases and 264 (74.2%) of the controls were SVD, whereas 39 (32.8%) of the cases and 47 (13.2%) of the controls were delivered by cesarean section (Fig. 2).

**Fig. 3:**
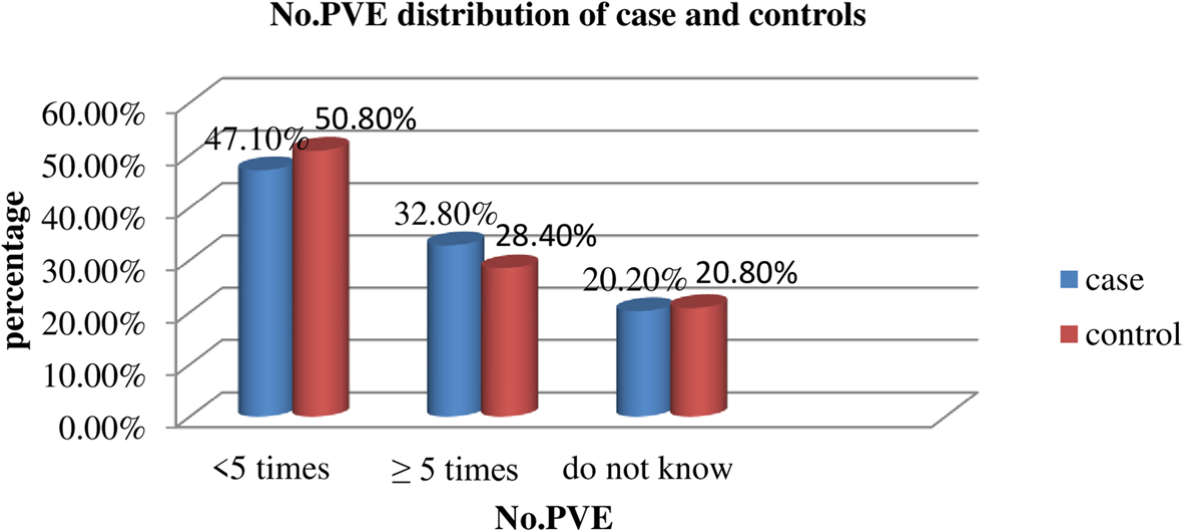
Frequency of per-vaginal examination postpartum women at Gedeo Zone public hospitals, Southern Ethiopia, 2023

Of the study participants, 39 (32.8%) cases and 101 (28.4%) controls had more than or equal to five times. Fifty-six (47.1%) of cases and 181 (50.8%) controls followed the number of per-vaginal examinations less than five times (Fig. 3).

**Fig. 4:**
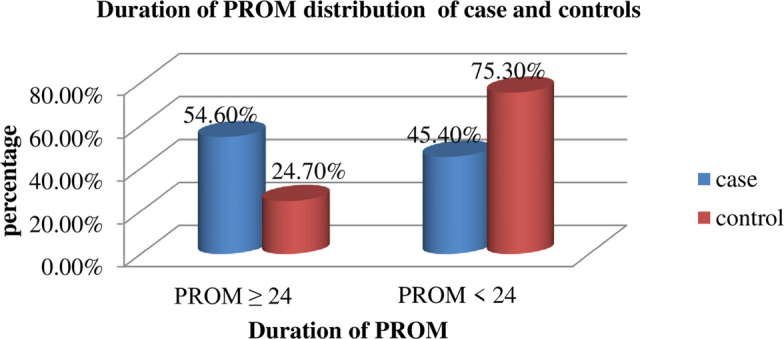
Duration of PROM of postpartum women at Gedeo zone public hospitals, Southern Ethiopia, 2023

When we see the duration of premature rupture of the membrane among the study participants, about 65 (54.6%) of the cases had PROM ≥ 24 h, and the majority (75.3%) of the controls had PROM < 24 h (Fig. 4).

### Medical or Co-morbidities of the mothers

With regards to the medical characteristics of the study participants, 12 (10.1%) of the cases and 19 (5.3%) of the controls had Gestational diabetes mellitus. Similarly, 53 (44.5%) of the cases and 92 (25.8%) of the controls were anemic (Table 4).

**Table 4:** Medical problems among postpartum mothers in Gedeo Zone at public hospital, 2023

Variable		Puerperal sepsis status		Total N (%)
		Case N (%)	Control N (%)	
HIV	yes	30 (25.2)	109 (30.6)	139 (29.3)
	no	89 (74.8)	247 (69.4)	336 (70.7)
GDM	yes	12 (10.1)	19 (5.3)	31 (6.5)
	no	107 (89.9)	337 (94.7)	444 (93.5)
STI	yes	45(37.8)	133(37.4)	178(37.5)
	no	74 (62.2)	223 (62.6)	297 (62.5)
Anemia status	Anemic	53 (44.5)	92 (25.8)	145 (30.5)
	Non anemic	66 (55.5)	164 (74.2)	330 (69.5)

*Abbreviation*: *GDM* Gestational diabetes mellitus, *STI* Sexually transmitted disease, *HIV* Human immune virus

### Health facility-related factors

More than half (53.8%) of cases and 222 (62.5%) of control women observed a person assisting them using only gloves. Around 31 (26.1%) of cases and 83 (23.3%) of control women observed a person assisting them in washing their hands and using gloves. Among the participants, 101 (84.9%) of cases and 333 (93.5%) controls had assisted during delivery. Out of them, 55 (54.5%) cases and 149 (44.7%) controls were delivered by health professionals (Table 5).

**Table 5:** Health-facility related characteristics of postpartum women in Gedeo Zone at public hospital, 2023

Variable		Puerperal sepsis status		Total N (%)
		Case N (%)	Control N (%)	
referred status	yes	47 (39.5)	133 (37.4)	180 (37.9)
	no	72 (60.5)	223 (62.6)	295 (62.1)
Assisted during delivery	Yes	101 (84.9)	333 (93.5)	434 (91.4)
	No	18 (15.1)	23 (6.5)	41 (8.6)
Birth attendant	health work	55 (54.5)	149 (44.7)	204 (47.0)
	TBA	46 (45.5)	184 (55.3)	230 (53.0)
hygienic practice	none	24 (20.2)	51 (14.3)	75 (15.8)
	used glove	64 (53.8)	222 (62.4)	286 (60.2)
	used gloves and washed hands	31 (26.1)	83 (23.3)	114 (24.0)

*Abbreviation*: *TBA* Traditional birth attendant, *ANC* Antenatal care

### Determinants of puerperal sepsis among postpartum women

In bi-variable logistic regression analysis, place of delivery, mode of delivery, ANC follow-up, Duration of premature rupture of membrane, Duration of labor, assisted delivery, gestational diabetes mellitus, obstructed labor, preeclampsia, postpartum hemorrhage, and anemia showed association with puerperal sepsis (*P* < 0.25). After applying multivariable logistic regression, mode of delivery, ANC follow-up, Duration of premature rupture of membrane, Duration of labor, postpartum hemorrhage, and anemia were associated (significant at *p* < 0.05) with puerperal sepsis (Table 6).

**Table 6:** Bivariable & multivariable logistic regression result for the determinants of puerperal sepsis among postpartum women at Gedeo Zone public hospitals, Southern Ethiopia, 2023

Variable		Puerperal sepsis status		OR (95% CI)		P-value
		Cases N (%)	Controls N (%)	COR(95%CI)	AOR(95%CI)	
Residence	urban	50 (42.0)	207 (58.1)	1	1	
	rural	69 (58.0)	149 (41.9)	1.92(1.26–2.92)	1.79 (.92–3.48)	0.068
ANC follow-up	yes	51 (42.9)	276 (77.5)	1	1	
	no	68 (57.1)	80 (22.5)	4.60(2.96–7.14)	4.19 (2.32–7.58)**	** < 0.001**
Duration of labor	< 12 h	6(5.0)	31 (8.7)	1	1	
	12–24 h	43 (36.1)	250(70.2)	.89(0.35–2.25)	.59 (0.19–1.78)	0.325
	≥ 24 h	70(58.8)	75 (21.1)	4.82 (1.89–12.25)	3.15 (1.09–9.43)*	**0.041**
PROM	< 24 h	54 (45.4)	268 (75.3)	1	1	
	≥ 24 h	65 (54.6)	88 (24.7)	3.66(2.37–5.65)	3.31 (1.89–5.83)**	** < 0.001**
Assisted during delivery	yes	101 (84.9)	333 (93.5)	1	1	
	no	18 (15.1)	23 (6.5)	2.58(1.34–4.97)	2.31 (0.88–6.06)	0.090
Mode of delivery	SVD	74 (62.2)	264 (74.2)	1	1	
	C/S	39 (32.8)	47 (13.2)	2.96 (1.80–4.87)	2.74(1.19–6.31)*	**0.018**
	Instrumental	6 (5.0)	45 (12.6)	.48 (0.19–1.16)	.37(0.12–1.15)	0.080
Place of delivery	home	29(24.4)	45 (12.6)	2.23(1.32–3.75)	.89 (.39–2.03)	0.748
	Institutional	90 (75.6)	311 (87.4)	1	1	
obstructed labor	Yes	19 (16.0)	29 (8.1)	2.14(1.15–3.98)	1.17(0.52–2.65)	0.629
	No	100 (84.0)	327 (91.9)	1	1	
PPH	No	71 (59.7)	298 (83.7)	1	1	
	Yes	48 (40.3)	58 (16.3)	3.47(2.19–5.51)	2.57 (1.39–4.74)**	**0.003**
preeclampsia	Yes	31 (26.1)	65 (13.2)	1.57 (0.97–2.57)	.94(0.49–1.82)	0.969
	No	88 (73.9)	291 (81.7)	1	1	
GDM	Yes	12 (10.1)	19 (5.3)	1.99(.93–4.23)	1.72 (0.67–4.44)	0.289
	No	107 (89.9)	337 (94.7)	1	1	
Anemia of mother	Non an	66 (55.5)	264 (74.2)	1	1	
	anemic	53 (44.5)	92 (25.8)	2.30(1.49–3.55)	2.46(1.39–4.32)**	**0.002**

^*^_= *p*- value < 0.05, **= *p*- value < 0.01, 1 = reference, *AOR* Adjusted Odd Ratio, *COR* crude odds ratio, and *CI* Confidence interval_

*Abbreviation*: *PROM* Premature rapture of the membrane, *ANC* Antenatal care, *PPH* Postpartum hemorrhage, *GDM* Gestational diabetes mellitus

In this study, the odds of developing puerperal sepsis among postpartum women with no ANC follow-up were 4.19 times higher than those of postpartum women who had attended ANC follow-up (AOR = 4.19; 95% CI: 2.32–7.58).

Accordingly, postpartum women who underwent a cesarean section had 2.74 times higher chances of developing puerperal sepsis compared to those who had a spontaneous vaginal delivery (AOR = 2.74; 95% CI: 1.19–6.31).

Mothers who were in labor for ≥ 24 h were 3.15 times more likely to develop puerperal sepsis compared with those < 12 h (AOR = 3.15; 95% CI: 1.09–9.43). Concerning the Duration of premature rupture of the membrane, mothers with PROM duration ≥ 24 h were 3.31 times more likely to develop puerperal sepsis than mothers with PROM duration < 24 h (AOR = 3.31; 95% CI: 1.89–5.83).

Postpartum hemorrhage was also another variable associated with puerperal sepsis, which indicated mothers who had postpartum hemorrhage were 2.57 times more likely to develop puerperal sepsis than those who had no postpartum hemorrhage (AOR = 2.57; 95% CI: 1.39–4.74).

When we see mothers' medical co-morbidity, the odds of developing puerperal sepsis among postpartum women with anemia were 2.46 times higher than those of postpartum women who had no anemia (AOR = 2.46; 95% CI: 1.39–4.32).

Table 6 Bivariable & multivariable logistic regression results for the determinants of puerperal sepsis among postpartum women at Gedeo Zone public hospitals, Southern Ethiopia, 2023.

## Discussion

This study aimed to identify the determinants of puerperal sepsis among postpartum women at a public hospital in the Gedeo Zone. According to this study, no ANC follow-up, cesarean delivery, PPH, duration of PROM, anemia, and Duration of labor ≥ 24 h were identified as the main determinants for puerperal sepsis.

In this study, puerperal sepsis was strongly associated with ANC follow-up; Mothers who had no ANC follow-up were 4.19 times more likely to be exposed to puerperal sepsis. This is in line with a study done in a case–control study in Uganda [7] and a cross-sectional study done in Kenya [32]. This may occur due to the identification and management of maternal conditions that increase the risk of puerperal sepsis, such as anemia, during ANC follow-up. However, if the mother did not receive ANC follow-up, as in the case mentioned in this study, mothers would be at risk of developing puerperal sepsis. This implies that the majority of pregnant women in low-resource settings are likely to miss out on important antepartum care schedules due to difficulties in accessing health facilities and cultural norms.

The result of this study also found that the mode of delivery had a significant association with the occurrence of puerperal sepsis. The odds of developing puerperal sepsis were 2.74 times higher among mothers delivered by cesarean than those by SVD. This finding was supported by a study conducted in the Oromia region of Ethiopia [25], Kenya [31], and Uganda [33]. This association may be explained by factors such as prolonged hospital stay, which can increase the risk of hospital-acquired infection, poor aseptic techniques in operating rooms, and poor hygiene practices of the women after the procedure. However, this finding contradicts a study conducted in the Gondar referral hospital in which women who gave birth by C/S were 2% less likely to develop puerperal sepsis than those who gave birth through SVD [21]. The variation in aseptic techniques employed in the study settings could account for this dissimilarity, while differences in methodology (such as study area, sample size, and study design) may also play a role in explaining this discrepancy.

The duration of the rupture of the membrane was also one of the other associated factors identified in this study. Mothers whose amniotic fluid membrane ruptured for more than 24 h were 3.31 times more likely to develop puerperal sepsis. This finding is in line with a similar study in the Oromia region of Ethiopia [25] and another study in Ethiopia [22]; this can be because prolonged PROM may facilitate the entrance of bacteria toward the pelvis and indirectly facilitate the development of puerperal sepsis. However, a study conducted in the USA stated that premature rupture of membranes was less likely to occur in those who eventually developed sepsis [34]. The difference may be that this study is facility-based, whereas the USA study is population-based. The variations might be related to health access, socio-cultural differences, the population under study, and awareness.

The duration of labor was significantly associated with puerperal sepsis. Mothers who were in labor for more than 24 h were 3.15 times more likely to develop puerperal sepsis than mothers who were in labor for less than 12 h. This finding is similar to the study conducted in the Oromia region of Ethiopia [25], Bahirdar, Ethiopia [20], the study done in Kenya [31], and Nigeria [35]. This may be because prolonged labor leads women to frequent vaginal examinations, perianal tears, or episiotomies and exposes them to an ascending infection. This implies that bacterial infection can be introduced by frequent vaginal examinations, prolonged rupture of membranes, obstructed labor, and tissue damage due to prolonged labor.

The odds of experiencing puerperal sepsis were 2.57 times more likely among mothers who had postpartum hemorrhage during delivery than those who had no PPH. The findings of this study were consistent with the studies conducted in China and India [36, 37] and Tanzania [8]. This might be due to placental abnormalities that could result in abnormal adherence of the placenta to the uterine endometrium, which is difficult to separate immediately after delivery of the fetus and makes the uterus lax, causing it to bleed a lot as well. This can result in uterine atony, which is a risk factor for postpartum hemorrhage [38]. However, a study conducted in California, United States of America, indicated that postpartum hemorrhage was not associated with puerperal sepsis [39]. The reason for these disagreements might be due to differences in the quality of health care service availability and accessibility that could help detect and manage postpartum hemorrhage during labor.

Anemia is also another significant risk factor identified in this study. Mothers who had anemia were 2.46 times more likely to have puerperal sepsis. The results of this study were in line with research done in Scotland that showed a substantial correlation between anemia and puerperal sepsis [40], China [37], and Tanzania [8]. This may be because anemia predisposes the development of different types of infections. The implication is that improper use of Iron-Folate and nutrition diversification during ANC follow-up caused anemia. However, those who had adequate use of Iron-Folate supplements and nutrition diversification during ANC follow-up should eliminate puerperal sepsis caused by anemia.

### Strength of the study

The strength of this study was that both controls and cases were selected from the same hospitals to reduce selection bias, and that it used patient interviews and medical record data to assess all variables.

In this study, new variables like obstructed labor, multiple pregnancies, preeclampsia, and post-term birth were added, and their significance was tested.

### Limitations of the study

This study has some limitations. First, there is a risk of recall bias since some of the information, such as obstetric history, is self-reported. Second, social desirability bias cannot be excluded, especially in questions regarding hygiene and health-seeking behaviour. Finally, because the study was conducted in selected hospitals, the findings may not be generalizable to all postpartum women in the community.

## Conclusion

The study found that lack of ANC follow-up, cesarean delivery, prolonged rupture of membranes ≥ 24 h, postpartum hemorrhage, prolonged labor ≥ 24 h, and maternal anemia were significantly associated with higher odds of puerperal sepsis. These findings indicate the need for improved ANC utilization, timely management of labor complications, strict infection-prevention practices during cesarean delivery, and strengthened prevention and treatment of anemia. Since this was a case–control study, the results show associations and should not be interpreted as causal.

### Recommendations

Based on the findings of this study, strengthening ANC follow-ups is highly recommended to ensure early detection and management of maternal conditions, including anemia. On the other hand, health facilities should equally improve the timely identification and management of both prolonged labour and prolonged rupture of membranes. Improved infection-prevention practices in cesarean delivery, appropriate labour monitoring by health workers guided through national protocols, and prevention/timely treatment of postpartum hemorrhage and anemia with better clinical management and nutrition counseling are recommended.

## Data Availability

We described all the necessary information in the publication, but the revised dataset can be received from the corresponding author upon reasonable request.

## Abbreviations

ANC: Antenatal Care
AOR: Adjusted Odds Ratio
CI: Confident Interval
C/S: Cesarean Section
COR: Crudes Odds Ratio
CSA: Central Statistical Agency
EDHS: Ethiopian Demographic and Health Survey
EMOH: Ethiopian Ministry of Health
GDM: Gestational Diabetic Mellitus
GZHD: Gedeo Zone Health Department
HIV: Human Immune-deficiency Virus
HSTP: Health Sector Transformation Plan
HTN: Hypertension
PPH: Postpartum Hemorrhage
SDG: Sustainable Development Goal
SNNPR: South Nations and Nationalities People Region
SPSS: Statistical Package for Social Science
SVD: Spontaneous Vaginal Delivery
UN: United Nation
WHO: World Health Organization

## References

[1] WHO, WHO recommendations for prevention and treatment of maternal peripartum infections. 2016: World Health Organization. 26598777

[2] WHO, U., UNFPA, World Bank Group, and the United Nations Population Division. Trends in maternal mortality 2000 to 2017: estimates by WHO, UNICEF. 2019, UNFPA, world bank group, and the United nations population division. Geneva ….

[3] Hussein J, et al. A review of health system infection control measures in developing countries: what can be learned to reduce maternal mortality. Glob Health. 2011;7(1):1–9. 10.1186/1744-8603-7-14 PMC3113713 21595872

[4] WHO, Statement on maternal sepsis. 2017, World Health Organization.

[5] Say L, et al. Global causes of maternal death: a WHO systematic analysis. Lancet Glob Health. 2014;2(6):e323–33. 25103301 10.1016/S2214-109X(14)70227-X

[6] Burchett HE, Mayhew SH. Maternal mortality in low-income countries: what interventions have been evaluated and how should the evidence base be developed further? Int J Gynaecol Obstet. 2009;105(1):78–81. 19201403 10.1016/j.ijgo.2008.12.022

[7] Ngonzi J, et al. Puerperal sepsis, the leading cause of maternal deaths at a tertiary university teaching hospital in Uganda. BMC Pregnancy Childbirth. 2016;16:1–7. 27495904 10.1186/s12884-016-0986-9 PMC4974713

[8] Kajeguka DC, et al. Factors and causes of Puerperal Sepsis in Kilimanjaro, Tanzania: A descriptive study among postnatal women who attended Kilimanjaro Christian Medical Center. The East African Health Research Journal. 2020;4(2):158. 34308233 10.24248/eahrj.v4i2.639 PMC8279318

[9] EMOH, Basic Emergency Maternal and Neonatal Care (BEMoNC). 2017.

[10] Mekonnen, W. and A. Gebremariam, Causes of maternal death in Ethiopia between 1990 and 2016: systematic review with meta-analysis. Ethiopian Journal of Health Development, 2018. 32(4).

[11] Atlaw D, et al. Puerperal sepsis and its associated factors among mothers in University of Gondar referral hospital, Ethiopia, 2017. International Journal of Pregnancy & Child Birth. 2019;5(5):190–5.

[12] Berhan, Y. and A. Berhan, Causes of maternal mortality in Ethiopia: a significant decline in abortion related death. Ethiop J Health Sci, 2014. 24 Suppl(0 Suppl): p. 15–28. 10.4314/ejhs.v24i0.3s PMC4249203 25489180

[13] Khaskheli MN, Baloch S, Sheeba A. Risk factors and complications of puerperal sepsis at a tertiary healthcare center. Pak J Med Sci. 2013;29(4):972–6. 24353670 10.12669/pjms.294.3389 PMC3817780

[14] López JP, Ángel-Müller E. Late puerperal sepsis, case report and literature review. Case Rep. 2016;2(1):17–26.

[15] WHO, WHO recommendations for prevention and treatment of maternal peripartum infections: highlights and key messages from the World Health Organization's 2015 global recommendations. 2015, World Health Organization. 26598777

[16] WHO, Global report on the epidemiology and burden of sepsis: current evidence, identifying gaps and future directions. 2020.

[17] CSA, Ethiopian demographic and health survey report. 2016.

[18] UN, The Sustainable Development Goals 2016. 2016, eSocialSciences.

[19] FMOH, E., Health Sector Transformation Plan II HSTP II. 2021.

[20] Admas A, et al. Proportion of bacterial isolates, their antimicrobial susceptibility profile and factors associated with puerperal sepsis among post-partum/aborted women at a referral hospital in Bahir Dar, Northwest Ethiopia. Antimicrob Resist Infect Control. 2020;9(1):14. 31956403 10.1186/s13756-019-0676-2 PMC6958633

[21] Atlaw D, Seyoum K. Puerperal sepsis and its associated factors among mothers in University of Gondar referral hospital, Ethiopia, 2017. Int J Pregnancy Childbirth. 2019;5(5):190–5.

[22] Melkie A, Dagnew E. Burden of puerperal sepsis and its associated factors in Ethiopia: a systematic review and meta-analysis. Arch Public Health. 2021;79(1):216. 34844656 10.1186/s13690-021-00732-y PMC8628469

[23] Tesfaye T, Samuel S, Lera T. Determinants of puerperal sepsis among postpartum women at public hospitals of Hawassa city, Southern Ethiopia: institution-based unmatched case-control study. Heliyon. 2023. 10.1016/j.heliyon.2023.e14809. 37025872 10.1016/j.heliyon.2023.e14809 PMC10070673

[24] GZHD, Gedeo Zone Health Department Demographic Statistics. 2022.

[25] Demisse GA, et al. Determinants of puerperal sepsis among postpartum women at public hospitals in West Shoa Zone, Oromia Regional State, Ethiopia (institutional case control study). BMC Pregnancy Childbirth. 2019;19(1):95. 30885159 10.1186/s12884-019-2230-x PMC6423770

[26] WHO, Managing prolonged and obstructed labor. World Health Organization, 2008.

[27] Organization, W.H., Diagnostic criteria and classification of hyperglycemia first detected in pregnancy. 2013, World Health Organization. 24199271

[28] Rockville, M., USA: CSA and ICF, Ethiopian Demographic and Health Survey. 2019: p. 199.

[29] Abebaw A, Gudayu TW, Kelkay B. Proportion of immediate postpartum anemia and associated factors among postnatal mothers in Northwest Ethiopia: a cross-sectional study. Anemia. 2020;2020:8979740. 32607255 10.1155/2020/8979740 PMC7315250

[30] Tamboli SS, Tamboli SB, Shrikhande S. Puerperal sepsis: predominant organisms and their antibiotic sensitivity pattern. Int J Reprod Contracept Obstet Gynecol. 2016;5(3):762–6.

[31] Naima, S., Magnitude and Risk Factors for Puerperal Sepsis at the Pumwani Maternity Hospital. 2017, University of Nairobi.

[32] Chepchirchir, M.V., J. Nyamari, and M. Keraka, Associated factors with Puerperal Sepsis among Reproductive Age Women in Nandi County, Kenya. Journal of Midwifery & Reproductive Health, 2017. 5(4).

[33] Ngonzi J, et al. Incidence of postpartum infection, outcomes, and associated risk factors at Mbarara regional referral hospital in Uganda. BMC Pregnancy Childbirth. 2018;18:1–11. 29954356 10.1186/s12884-018-1891-1 PMC6022296

[34] Al-Ostad G, et al. Incidence and risk factors of sepsis mortality in labor, delivery, and after birth: population-based study in the USA. J Obstet Gynaecol Res. 2015;41(8):1201–6. 25976287 10.1111/jog.12710

[35] Ononuju C, et al. Risk factors and antibiogram of organisms causing puerperal sepsis in a tertiary health facility in Nigeria. Tropical Journal of Obstetrics and Gynecology. 2015;32(2):73–82.

[36] Karsnitz DB. Puerperal infections of the genital tract: a clinical review. J Midwifery Womens Health. 2013;58(6):632–42. 24406036 10.1111/jmwh.12119

[37] Song H, et al. Risk factors, changes in serum inflammatory factors, and clinical prevention and control measures for puerperal infection. J Clin Lab Anal. 2020;34(3):e23047. 31883276 10.1002/jcla.23047 PMC7083398

[38] da Cunha Castro EC, Popek E. Abnormalities of placenta implantation. APMIS. 2018;126(7):613–20. 30129132 10.1111/apm.12831

[39] Acosta CD, et al. The continuum of maternal sepsis severity: incidence and risk factors in a population-based cohort study. PLoS One. 2013;8(7):e67175. 23843991 10.1371/journal.pone.0067175 PMC3699572

[40] Acosta C, et al. Maternal sepsis: a Scottish population-based case–control study. BJOG Int J Obstet Gynaecol. 2012;119(4):474–83. 10.1111/j.1471-0528.2011.03239.x PMC3328752 22251396

